# Development of a UPLC-FLD Method for Detection of Aflatoxin B1 and M1 in Animal Tissue to Study the Effect of Curcumin on Mycotoxin Clearance Rates

**DOI:** 10.3389/fphar.2017.00650

**Published:** 2017-09-14

**Authors:** Xiaoxu Cui, Ishfaq Muhammad, Rui Li, Huiran Jin, Zhaolin Guo, Yuqi Yang, Sattar Hamid, Jiarui Li, Ping Cheng, Xiuying Zhang

**Affiliations:** ^1^Faculty of Basic Veterinary Science, College of Veterinary Medicine, Northeast Agricultural University Harbin, China; ^2^Heilongjiang Institute of Veterinary Drug and Feed Control Harbin, China

**Keywords:** UPLC-FLD, aflatoxin B1, aflatoxin M1, curcumin, arbor acres broiler, clearance time

## Abstract

Aflatoxin B1 (AFB1) and its metabolite aflatoxin M1 (AFM1) are well-known carcinogens for humans and animals health. In this study, an ultra-high performance liquid chromatography linked with fluorescence detection (UPLC-FLD) method was optimized and validated. In addition, we investigated for the first time, the influence of curcumin on residue depletion of AFB1 and AFM1 in liver, kidney, and muscle tissues of broiler chickens and estimated a necessary clearance time required for AFB1 and AFM1 residues. The results showed that the average recoveries of AFB1 varied in liver, kidney, and muscles between 82.32–85.56, 85.34–88.45, and 84.88–89.73% respectively, while the average recoveries of AFM1 in liver, kidney, and muscles varied between 92.17–95.03, 94.12–97.21, and 95.32–98.51%, respectively. The detection limit of aflatoxin B1 was 0.008 ng/ml, while for aflatoxin M1 was 0.003 ng/ml. The limit of quantification (LOQ) for AFB1 and AFM1 was 0.02 and 0.01 ng/ml, respectively. Clearance time for AFB1 and AFM1 residues were analyzed in two experimental groups of broilers. One group fed with dietary AFB1 (5.0 mg/kg feed) and other with curcumin+AFB1 diet (curcumin; 300 mg/kg feed, AFB1; 5.0 mg/kg feed). AFB1 and AFM1 residues clearance time was calculated based on LOQ using withdrawal time calculation software (WT1.4). Clearance time analyzed for AFB1 ranged from 11 to 19 days and for AFM1 ranged from 10 to 12 days at 95% confidence level. Interestingly, curcumin supplementation in the diet reduced the clearance time of AFM1 in liver and kidney but not in muscle tissues. Conclusively, the developed method can be appropriately used for the quality control testing of commercial broiler-meat processing companies, food manufacturers, and quality control laboratories.

## Introduction

Two *Aspergillus* species known as *Aspergillus flavus* and *Aspergillus parasiticus* produces B and G aflatoxins. These *Aspergillus* species mainly found in regions with hot and humid climates. B and G aflatoxins have been reported earlier in a variety of foods and feed stuffs such as oil seeds, cocoa, dried peas, nuts, pistachios, beans, etc. All of these toxins including the metabolite (AFM1) of Aflatoxin B1 (AFB1) are considered carcinogenic and harmful for humans (humans carcinogenic, Group 1, IARC) (Saini and Kaur, 2012). Moreover, AFB1 causes immunosuppression (Hussain et al., 2010), hepatotoxicity, genotoxicity, and produces other harmful effects in many animal species including poultry (Richard, 2007). AFM1 (hydroxylated metabolite) present in animal products that eat foods contaminated with AFB1 toxin, classified in Group 2B carcinogens (Muhammad et al., 2017), cytotoxic in nature and causes similar toxic effects to that of AFB1 (André et al., 2011). Previous studies reported a major loss of meat/egg production, immunosuppression and hepatotoxicity in acute or chronic aflatoxicosis in poultry birds (Verma et al., 2004; Khan et al., 2010). Aflatoxin (AF) residues in edible tissues such as kidney, liver and muscles tissues were detected in poultry birds fed aflatoxin (AF) contaminated diet under experimental conditions. In addition, AF residues have also been determined in samples collected from commercial poultry farms (Bintvihok and Davitiyananda, 2002). Apart from hazard to the poultry industry and livestock, aflatoxins causes a serious public health hazard, responsible for chronic and acute liver failure and Reyes syndrome (Yaqi et al., 2017). Aflatoxin B1 (Group 1 human liver carcinogen) acts synergistically with hepatitis B virus (HBV) to enhance 12-fold the risk of liver cancer. Moreover, aflatoxins is a causative factor in child mortality, underweight, neurological impairment, and immunosuppression (World Health Organisation, 2006). Previously, a Commission Regulation set the maximum legal limits for aflatoxins and its residues for all the European Union (EU) countries for a number of food/feed products (Commission Regulation, 2001). However, the EU has not provided MLs for aflatoxin in meat and meat products. Based on the above mentioned concerns, it is imperative to establish an accurate, sensitive and effective method for the detection of aflatoxin residues; to ensure the safety of livestock and poultry products and maintain human health.

Curcumin (*Curcuma longa* Linn.) is a hydrophobic polyphenol derived from turmeric, commonly called diferuloylmethane. Commercial curcumin composed of a mixture of curcuminoids; bisdemethoxycurcumin, demethoxycurcumin, and diferuloylmethane (Anand et al., 2008). It has been demonstrated earlier that the carbonyl functional group of curcuminoids shown to be responsible for its therapeutic (anti-carcinogenic and anti-mutagenic) actions (Chun et al., 1999). In a previous study, curcumin was used as a chelator to reduce the affinity of Al^3+^ on DNA binding (Ahmadi et al., 2011). Furthermore, curcumin is highly effective against AFB1 toxicity (Soni et al., 1997), protect the liver from AFB1 harmful effects, and oxidative damage by preventing the biotransformation of AFB1 in liver (Lee et al., 2001). However, to the best of our knowledge, the influence of curcumin on AFB1 and AFM1 residues depletion and the clearance of these residues from the chicken edible tissues after the withdrawal of dietary AFB1 have not been adequately studied. Therefore, we employed curcumin in current study to investigate its effect on AFB1 and AFM1 residues depletion in liver, kidney, and muscles of broilers.

To our knowledge, thin-layer chromatography (TLC) was used for the determination and quantitation of aflatoxins for several decades. Recently, high performance liquid chromatography (HPLC) is a method of choice due to its short and high-resolution columns, together with the sensitivity of fluorescence detectors. In addition, most of the previous and recent studies used immunoaffinity columns (IAC) cleanup for the analysis of aflatoxins and its residues in a variety of food/feed-products (Holcomb et al., 1992; Valenta, 1998). In this study, an IAC cleanup step was employed to develop an accurate, sensitive, rapid, and improved method for the detection of AFB1 and AFM1 residues. Considering the toxicity of AFB1 (most dangerous among aflatoxins) and its foremost metabolite AFM1, and the use of broilers meat by consumers concerned with health, it is necessary to have a reliable, sensitive, and quick methodology for measuring AFB1 and AFM1 residue level in broiler tissues. In this study, we optimized and validated for the first time an improved analytical method using ultra-high performance liquid chromatography linked with fluorescence detection (UPLC-FLD) for the identification and quantitation of AFB1 and AFM1 residues in liver, kidney and muscle tissues of broilers by using immunoaffinity column with quick and high test recovery as compared with other intended procedures of analysis. In addition, the developed method is applied to study the influence of curcumin on depletion of AFB1 and AFM1 residues and to measure the duration of clearance time of the above two carcinogens from the liver, kidney, and muscle tissues of broilers maintained on AFB1-contaminted feed.

## Materials and methods

### Apparatus

Ultra High Performance Liquid Chromatography Acquity (UPLC, USA) with a Fluorescence Detector (FLD, Waters, USA) was used for measurement. Aflatoxin immunoaffinity columns (AFM1 immunoaffinity column; Hua anmai, Company Beijing and AFB1 immunoaffinity column; VICAM, USA) was used for immunoaffinity cleanup step. High-speed rotating grinder (Fritsch Corporation, Germany), Pumping operation frame (Agilent), N-EVAPTM112 Termovap instrument (American Organomation Associates), MS3 type swirling oscillator (IKA Corporation), pH meter (METTLER TOLEDO Instrument company Ltd.), MP2002 electronic analytical balance, SHZ-C-type constant-temperature water bath oscillator (Pudong Physical Optical Instrument Factory, Shanghai, China) were used for sample preparation and extraction.

### Reagents and chemicals

All reagents and chemicals used were of analytical grade. AFB1 (purity ≥99.0%) and AFM1 (0.5 μg/ml) was bought from Sigma-Aldrich (St. Louis, USA). Experimental procedures were performed with MilliQ water (deionized) provided by Youpu company, Ltd. (Sichuan, China). NaCl, KCl, Na_2_HPO_4_.12H_2_O, and KH_2_PO_4_ purchased from Kemi chemicals company Ltd. (Tianjin, China). Acetonitrile (99.9%) and methanol (99.9%) of HPLC grade obtained from Fisher Company (USA).

### Standard working solutions

One milligram of AFB1 was dissolved in 10 ml of methanol in a volumetric flask to get AFB1 stock solution of 100 μg/ml. One milliliter of AFM1 (0.5 μg/ml) was diluted in 10 ml of acetonitrile in volumetric flask to obtain stock solution of 0.05 μg/ml. The AFB1 and AFM1 standard solutions were stored at −20°C. The two standard solutions were then diluted with the corresponding solvents before use to the desired concentrations of AFB1 (0.0080, 0.0100, 0.0200, 0.1000, 0.4000, and 1.0000 ng/ml) and AFM1 (0.0030, 0.0080, 0.0200, 0.1000, 0.4000, and 1.0000 ng/ml), and stored at 4°C not more than 30 days. The calibration curves drawn for AFB1 and AFM1 were based on the above six level concentrations. AFB1 and AFM1 both are toxic and liver carcinogens, therefore it should be handled with great care to avoid contact/inhalation of the chemicals and should only be handled in a fume hood while wearing gloves, lab coat, and goggles (to protect eyes). All contacted materials and the toxic chemicals (AFB1 and AFM1) itself, should be properly disposed of in a licit and environmentally safe manner.

### Experimental chickens and sample collection

Hundred and twenty 1-day-old commercial Arbor Acres broiler chickens were bought from Yi Nong Commercial hatchery (Heilongjiang, China, registration number; 230108799294096). The chickens were divided into three groups. Forty chickens were allocated randomly in each group; Group 1; fed normal diet, Group 2; fed on 5.0 mg AFB1/kg contaminated diet and Group 3; fed on curcumin plus AFB1 supplemented diet (300 mg curcumin/kg and 5.0 mg AFB1/kg diet). The chickens were maintained on 12 h light and 12 h dark and feed provided *ad libitum*. AFB1 was mixed with feed by dissolving 10 mg of AFB1 in 30 ml methanol. Then, the AFB1-methanol solution was sprayed over the feed and mixed to attain the required concentration of AFB1 (5.0 mg AFB1/kg feed), and finally the feed air-dried at 37°C. The group 1 chickens were euthanized and sacrificed on day 14, and the liver, kidney, and muscle tissues were collected and stored at −80°C. After 28 days, group 2 and group 3 chickens were fed on normal basal diet up to 42 days. Six chickens from each group were sacrificed following euthanasia on day 28, 30, 32, 35, 38, and 42. The liver, kidney, and muscle tissues were collected and immediately stored at −80°C until further analysis. The experiments were conducted under the supervision of the Harbin Veterinary Research Institute of the Chinese Academy of Agricultural Sciences in accordance with animal ethics guidelines and approved protocols [SYXK (Hei) 2012-2067].

### Sample preparation and immunoaffinity column clean-up

The immunoaffinity column cleanup was simple and quick. It has been demonstrated previously that the use of immunoaffinity clean-up step provide a highly sensitive method for determination of aflatoxins and avoiding the use of substantial amounts of toxic and dangerous chemicals compared to conventional clean-up (Chiavaro et al., 2005). A tissue sample (from group 1; fed normal diet) weighing 2 ± 0.02 g was first ground with a tissue grinder machine (Fritsch Corporation) and spiked with AFB1 and AFM1. While, AFB1 and AFM1 extractions from liver, kidney and muscle tissue of group 2 and group 3 samples were performed according to the AOAC guidelines (AOAC International, 1995, Chap. 49) (AOAC International, 1995). Twenty milliliters of dichloromethane was added to the spiked sample and the mixture was ultrasonicated for 10 min and shaken continuously for 1 h on a shaker to assist extraction. Anhydrous sodium sulfate (2 g) was added and centrifuged at a speed of 10,000 rpm for 5 min. Finally, 10 ml of supernatant was collected and dried in Termovap instrument (American Organomation Associates) in a water bath at 50°C. The clean-up steps were performed according to the guidelines stated in previous study (R-Biopharm, Rhône, 2001). In brief, 2 ml of methanol and 13 ml of PBS was added to the residue and dissolved well. Next, the elution was carried out with 1 ml methanol. The solution passed through immunoaffinity column (IAC) at a rate of 1–2 drops/s. The column was washed with 10 ml deionized water and the whole eluate was dried in Termovap instrument in a water bath at 50°C. The remaining residue placed in water bath at 40°C for 15 min, and 100 μl of trifluoroacetic acid and 200 μl of hexane was added to it and dried again by a gentle stream of nitrogen at 50°C. Finally, the quantitation of AFB1 and AFM1 residues level was detected by UPLC-FLD.

### UPLC-FLD analysis

AFB1 and AFM1 was determined by Ultra High Performance Liquid Chromatography (UPLC) Acquity (Waters, USA), equipped with a Fluorescence Detector (FLD, Waters, USA). Chromatographic column (Waters Acquity UPLC BEH C18) employed in the experiments with the size (1.7 μm × 2.1 mm × 50 mm) for the separation of analytes, the flow rate maintained through the column was 0.2 ml/min, and the injection volume was 10 μl. The mobile phase consisted of acetonitrile: water (20:80) and calibration curves were based on the analysis of working solutions for AFM1 (0.0030, 0.0080, 0.0200, 0.1000, 0.4000, and 1.0000 ng/ml) and AFB1 (0.0080, 0.0100, 0.0200, 0.1000, 0.4000, and 1.0000 ng/ml), respectively. The excitation and emission wavelength was 360 and 435 nm, respectively. The system was computer controlled and EMPOWER3 software was used for the analysis of data.

### Method validation

In order to verify the performance and validation characteristics of the method, we followed the guidelines of Brazilian Institute of Metrology, Quality and Technology (Camargo et al., 2011; INMETRO, 2011), and evaluated the parameters such as limits of detection (LOD) and quantification (LOQ), sensitivity, selectivity, robustness, accuracy (recovery), linearity, and precision (repeatability and intermediate precision) of the method. The limit of detection (LOD) and limit of quantification (LOQ) was detected based on signal-to-noise ratio (S/N). The accuracy of the method was determined by evaluating percent recoveries of AFB1 and AFM1 residues. A known concentration of analytes (0.0100, 0.0200, and 0.0400 μg/kg for AFB1 and 0.0050, 0.0100, and 0.0200 μg/kg for AFM1) were added to blank matrix samples for recovery experiments. The linearity was determined using solutions prepared from the standard solutions, in three replicates, of six levels of concentrations of the AFB1 and AFM1 standards, over the range of 0.0080–1.0000 ng/ml for AFB1 and 0.0030–1.0000 ng/ml for AFM1, respectively. The repeatability of the method was calculated by coefficient of variation (%RSD). A single analyst performed all the above analysis by using the same equipment.

### Statistical analysis

The method mentioned was optimized and developed for all steps with statistical treatments that increase the test recovery, save time and reagents, and lessen the matrix interferences. AFB1 and AFM1 peak was chromatographically separated well. Linear regression and significance (*p* < 0.05 or *p* < 0.01) of the data was analyzed by ANOVA using SPSS (Version 17.0, USA) software Withdrawal Time software (WT 1.4) was used for the determination of clearance time at 95% confidence level (*p* < 0.05) (Committee for Veterinary Medicinal Products, 1996).

## Results and discussion

### Optimization of the UPLC-FLD parameters

AFB1 and AFM1 residues have been reported in previous studies (Fernandez et al., 1994; Khan et al., 2013). The requirements for accurate, sensitive, and reliable methods have led to a great advancement in the development of highly sensitive, selective, and accurate methods for the detection of AFB1 and AFM1 residues in chicken edible tissues. Thus, a rapid, robust, and reliable UPLC-FLD method was developed in this study for the simultaneous determination of AFB1 and AFM1 residues in broiler tissues. The method is novel, sensitive, and accurate in the sense that simultaneous determination of AFB1 and AFM1 residues in liver, kidney, and muscle tissues has been performed for the first time using immunoaffinity column in comparison with other reported laborious, less sensitive, complex, and expensive methods (Potesil et al., 2005; Petrlova et al., 2006; World Health Organisation, 2006; Han et al., 2010; Sebaei et al., 2012; Decleer et al., 2016). The following tests were performed to optimize the method according to the standard guidelines (Epshtein, 2004) and choose the most appropriate UPLC-FLD conditions.

#### Selection of extraction solvent and effect of shaking time on recovery

Sample preparation commonly plays a key role in the quality of chromatographic results. Samples properly prepared gives results free from interfering peaks. Previously, it has been stated that polar solvents are the efficient solvents used for extracting mycotoxins and water provides higher extraction efficiencies in mixtures, by enhancing penetration of the solvent (Hinojo et al., 2006; Sebaei et al., 2012). In this study, different extraction solvents were tested to facilitate the extraction of AFB1 and AFM1 residues from broiler tissues, however, dichloromethane was selected as the best extraction solvent due to its highest recoveries for both AFB1 and AFM1 residues (Table 1). The data clearly showed that the extraction yield and recovery rate was higher with the use of dichloromethane as compared to other solvents. Importantly, it should be kept in mind that dichloromethane is carcinogenic to experimental animals and possibly to humans and included in group 2B carcinogens. It causes skin and eye irritation, and can be absorbed into the body through skin, ingestion or by inhalation (https://pubchem.ncbi.nlm.nih.gov/compound/dichloromethane#section=Cancer-Risk). In order to optimize the efficient extraction conditions, the effect of shaking time for the highest recoveries of AFB1 and AFM1 were also tested. Supplementary Figure 1 showed that a minimum of 60 min shaking time in extraction procedure gives maximum AFB1 and AFM1 recovery values.

**Table 1:** Effect of extraction solvent on recovery (%) of AFB1 and AFM1.

**Name**	**100% Dichloromethane**	**70% Methanol**	**80% Methanol**	**90% Methanol**	**Methanol**	**84% Acetonitrile**
AFB1	88.55 ± 2.76	80.12 ± 3.45^a^	82.31 ± 2.58^a^	79.91 ± 3.91^a^	82.12 ± 4.21^a^	79.12 ± 3.25^a^
AFM1	95.42 ± 3.12	88.46 ± 2.98^a^	89.12 ± 4.02^a^	83.12 ± 3.26^a^	81.13 ± 3.78^a^	86.15 ± 3.69^a^

Each value is the mean of five replicates represented as mean ± SD (X ± SD; n = 5), lowercase

“a” *represents P < 0.01*.

#### Optimization of the chromatographic UPLC-FLD conditions

Recently, it has been demonstrated that the use of UPLC has plenty of advantages, increasing resolution, improving laboratory yield because of the brief and short analysis time, and mainly reducing solvent use and costs, compared to HPLC (Purcaro et al., 2013). The various chromatographic conditions such as column temperature, excitation, and emission wavelength, flow rate, and mobile phase composition were optimized for analysis of AFB1 and AFM1 residues using UPLC-FLD system and the method was appropriate to detect the above residues. The suitable temperature (25°C) was chosen to carry out experiments after testing different column temperatures (20, 25, 30°C). The results (Table 2) showed that by using 25°C, peak area and peak shape was good, and significantly (*P* < 0.01 or *P* < 0.05) different from the other tested temperatures (20 and 30°C). A range of excitation (300–400 nm) and emission wavelengths (390–400 nm) were tested for the detection of AFB1 and AFM1 residues respectively, and the excitation and emission wavelengths was set at 360 and 435 nm, respectively, due to its fit peak areas. An optimum flow rate of 0.2 ml/min was selected at a column temperature of 25°C due to obvious and better peak area and shape of chromatogram as compared to 0.1 and 0.3 ml/min flow rate. The peak is lower and peak-time is too late at a flow rate of 0.1 ml/min, while the peak area was not good at a flow rate of 0.3 ml/min indicated in Table 2. The mobile phase composition is very important because it mainly influences the peak response and retention of analytes (Khan et al., 2011). In order to select the most appropriate mobile phase ratio, a range of mobile phase ratio consisted of acetonitrile: water (20:80, 15:85, 25:75, and 30:70) were tested at a flow rate of 0.2 ml/min, an injection volume of 10 μl and at a column temperature of 25°C, and we selected the most optimum mobile phase ratio of acetonitrile: water (20:80) due to its good peak area and peak shape for the analysis of the studied residues, as shown in Table 3.

**Table 2:** Effect of flow rate and column temperature on peak area of AFB1 and AFM1.

**Name**	**0.1 ml/min**	**0.2 ml/min**	**0.3 ml/min**
**EFFECT OF FLOW RATE ON PEAK AREA OF AFB1 AND AFM1**			
AFB1	44,755 ± 1,419^a^	48,306 ± 1,665	43,128 ± 2,099^a^
AFM1	12,566 ± 1,316^a^	14,280 ± 711	11,298 ± 825^a^
**Name**	**20**°**C**	**25**°**C**	**30**°**C**
**EFFECT OF COLUMN TEMPERATURE ON THE AREA OF AFB1 AND AFM1**			
AFB1	42,755 ± 1,123^a^	48,306 ± 1,665	46,128 ± 1,045^a^
AFM1	11,536 ± 1206^a^	14,280 ± 711	10,298 ± 861^a^

Each value is the mean of five replicates of peak area calculated by empower 3 software represented as mean ± SD (X ± SD; n = 5), lowercase

‘a’ *represents P < 0.01*.

**Table 3:** Effects of mobile phase [acetonitrile:water (v/v)] on peak area of AFB1 and AFM1.

**Name**	**20:80**	**15:85**	**25:75**	**30:70**
AFB1	48,306 ± 1,665	43,705 ± 1819^a^	45,981 ± 1,494^A^	45,084 ± 1,416^a^
AFM1	14,280 ± 711	12,266 ± 1,046^a^	12,380 ± 1,157^a^	11,994 ± 837^a^

Each value is the mean of five replicates of peak area obtained by empower 3 software represented as mean ± SD (X ± SD; n = 5), capital letter

“A” indicates P < 0.05 and lowercase

“a” *represents P < 0.01*.

#### Method validation

Our results (limits of detection and quantification, accuracy, linearity, retention time, precision, coefficient of variation, and test recovery) showed that the UPLC-FLD method was fully validated and reliable for the determination of AFB1 and AFM1 residues in chicken edible tissues by using fluorescence detection. Supplementary Figures 2A–D showed that complete separation of AFB1 and AFM1 peaks were achieved, by analyzing the standard solution and spiked AFB1 and AFM1 liver, kidney and muscle tissues samples.

#### LOQ and LOD

The sensitivity of method was determined by LOD and LOQ for AFB1 and AFM1. It is well-understood that the minimum concentration of an analyte that can be detected is known as the LOD, while the lowest amount of an analyte in a sample that can be quantitatively determined with suitable precision is the LOQ. In a previous study, the LOD and LOQ was determined as “the concentration that produces a detector signal that could be easily known from the baseline (three times larger than the baseline noise)” and the LOQ as “the concentration that gives a detector signal 10 times larger than the baseline noise” (Stachniuk et al., 2016). In our study, we followed the above guidelines, and the signal-to-noise ratio (S/N) determined for detection limit (LOD) is equal to 3:1 and for the LOQ was 10:1.

### Method linearity

The level of linearity of the calibration curve is crucial for the quality of the tested method. Therefore, we followed an appropriate regression model, preferably a linear regression model (Van Loco et al., 2002). The linearity of the current method was assessed from the calibration curves obtained from standard working solutions of six different concentrations for AFM1 and AFB1, respectively. The coefficients of determination (*R*^2^) were calculated from the regression equations (AFB1; y = 2E+06x-2670.2 and AFM1; y = 1E+06x+5032.2) give high values (AFB1; 0.9998 and AFM1; 0.9997), revealed good linearity within the selected range for both AFB1 and AFM1 residues.

#### Selectivity

The ability to separate analytes from other component that may be present in the sample including impurities is known as selectivity of the method (Bliesner, 2005). Method selectivity is of major importance in validation, our proposed method (UPLC-FLD) allows the quantification of AFB1 and AFM1 residues simultaneously, and these residues do not interfere with each other. The UPLC fluorescence detection differentiate the AFB1 and AFM1 co-eluting peaks simultaneously and minimizing background influence. The chromatograms of liver, kidney and muscle tissues analyzing samples (spiked with AFB1 and AFM1) and standard chromatograms with the above stated UPLC-FLD conditions generated AFB1 and AFM1 reasonably resolved peaks displayed in Supplementary Figures 2A–D, showed good separation ability and selectivity of the developed method.

#### Accuracy

Accuracy is defined as “the closeness of agreement between an accepted reference/conventional true value and the value found.” The accuracy was determined by percent recoveries of analytes at three different concentrations (low, medium, and high) in liver, kidney, and muscle tissues of broiler chickens (Table 4). The average recoveries of five replicates of aflatoxin B1 at low, medium, and high concentration level ranged in liver, kidney and muscles between 82.32–85.56, 85.34–88.45, and 84.88–89.73% respectively, while the average recoveries of aflatoxin M1 in liver, kidney, and muscles of broiler chickens of five replicates ranged between 92.17–95.03, 94.12–97.21, and 95.32–98.51%, respectively (Table 4).

**Table 4:** Mean recovery (%) and precision (%RSD) (intra-day precision and inter-day precision) of AFB1 and AFM1 (*n* = 5) in liver, kidney, and muscles tissues.

**Name**	**Intra-day precision (%RSD)**			**Inter-day precision (%RSD)**			**Recovery (%)**		
	**Low**	**Medium**	**High**	**Low**	**Medium**	**High**	**Low**	**Medium**	**High**
**LIVER**									
AFB1	4.17	4.45	3.03	1.47	2.81	2.56	82.32 ± 3.49	83.21 ± 3.70	85.56 ± 2.58
AFM1	3.46	2.51	3.19	3.42	2.62	1.83	92.17 ± 3.19	95.03 ± 2.38	94.48 ± 3.01
**KIDNEY**									
AFB1	3.54	3.23	2.89	3.01	1.12	3.45	85.34 ± 3.02	86.19 ± 2.78	88.45 ± 2.56
AFM1	2.85	3.81	2.61	1.72	2.85	1.47	94.12 ± 2.68	95.78 ± 3.65	97.21 ± 2.54
**MUSCLE**									
AFB1	2.39	4.17	2.58	3.01	2.01	3.68	88.74 ± 2.13	89.73 ± 3.74	84.88 ± 2.19
AFM1	3.97	2.75	2.65	2.31	3.28	1.96	96.32 ± 3.84	95.32 ± 2.62	98.54 ± 2.61

*Each value is the mean of five replicates of percent recovery represented as mean ± SD (X ± SD; n = 5). The precision relative standard deviation (%RSD) calculated at three different levels of AFB1 concentrations. AFB1 concentrations level (Low = 0.0100 μg/kg, Medium = 0.0200 μg/kg, and High = 0.0400 μg/kg) and AFM1 concentrations level (Low = 0.0050 μg/kg, Medium = 0.0100 μg/kg and High = 0.0200 μg/kg*.

#### Precision and robustness of the method

Validation of methods for analytical measurements includes the analysis of precision and robustness. Precision was investigated at two levels: intermediate precision (inter-day) and repeatability (intra-day) and coefficient of variation (%RSD) was calculated by the Equation (1). (Peak area of Sample/peak area of Standard) × 100% (1) The precision statistics of the method is presented in Table 4 by coefficient of variation (%RSD) for low, medium, and high concentration levels within 1 day and to demonstrate the intermediate precision (between different days), the procedure was repeated on three consecutive days. The repeatability (intra-day precision) for AFB1 and AFM1 ranged from 2.39 to 4.45 and 2.51 to 3.97%, respectively, and intermediate precision (inter-day) for AFB1 and AFM1 was shown to be 1.12–3.68 and 1.47–3.42%, respectively (Table 4). These values are in agreement (< 20%) with EU guideline 96/23/EC (European Commission, 2002), and in agreement (< 15%) with FDA guideline (FDA, 2012) for a validated analytical method. The robustness of the method was evaluated by bringing slight changes in chromatographic conditions such as mobile phase ratio, column temperature, excitation and emission wavelength, and flow rate resulted negligible changes in the peak area of the analytes, proved that the method is robust, rugged, and fit. Thus, it is suggested that method can be easily applied for the assessment and determination of AFB1 and AFM1 residues in edible tissues of broiler chickens.

### Application of the method

Dietary ingestion of aflatoxins especially AFB1 causes many human health problems such as acute aflatoxicosis, hepatocellular cancer, immunosuppression, increased susceptibility to hepatitis B virus infection and growth abnormalities in different parts of the world particularly in African and Asian countries (Wild and Gong, 2010). Therefore, in this study, we evaluated clearance time and the impact of curcumin on AFB1 and AFM1 residues in broilers to safeguard human health against the most danger aflatoxin AFB1 and its metabolite AFM1. We applied a more reliable and sensitive method (UPLC-FLD) which have certain advantages such as increasing resolution, improving laboratory yield and short analysis time compared to HPLC (Purcaro et al., 2013). Furthermore, immunoaffinity columns have been used for more efficient extraction of AFB1 and AFM1 residues and avoiding the use of toxic chemicals utilized in conventional clean-up.

#### Proposed clearance time for AFB1 and AFM1 residues in liver, kidney, and muscle tissues of broilers

It is well-known that AFB1 and its foremost metabolite AFM1 are carcinogenic in nature. Hence, it is of vital importance to propose an appropriate clearance time for these toxic residues from chicken's edible tissues to prevent human health. To our knowledge, there is no fixed maximum limit (MLs) for AFB1 and AFM1 residues in chicken's meat. In order to safeguard human health, we used withdrawal time calculation programme (WT 1.4) to calculate necessary clearance time for AFB1 and AFM1 residues in liver, kidney, and muscle tissues of broilers based on LOQ. In calculation, if the clearance time did not make up a full day, it is rounded up to the next day (for example, 11.34–12 day).

##### Clearance time based on LOQ

**Figure 1:**
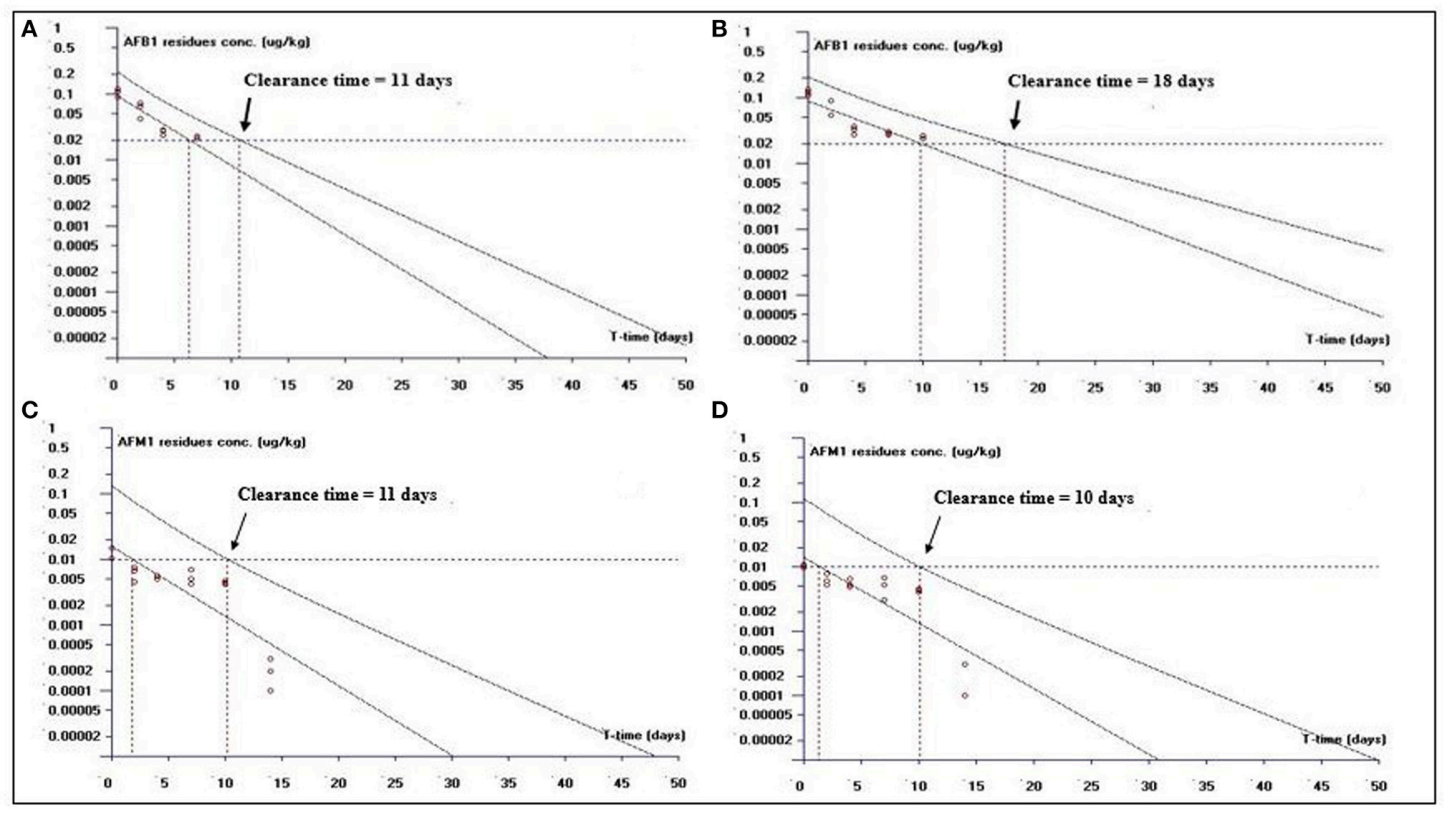
The representative semi-logarithmic plot shows aflatoxin B1 (AFB1) and aflatoxin M1 (AFM1) residue concentrations for broiler **liver tissues vs. time**, with the one-sided 95% upper tolerance limit in **AFB1-fed group; (A)** AFB1 residues **(C)** AFM1 residues and in **curcumin**+**AFB1-fed group; (B)** AFB1 residues **(D)** AFM1 residues. Small circles represent the residue concentrations of AFB1 and AFM1 for individual broiler chickens. The clearance period calculation based on the limit of quantification (LOQ; 0.02 μg/kg for AFB1 and 0.01 μg/kg for AFM1).

**Figure 2:**
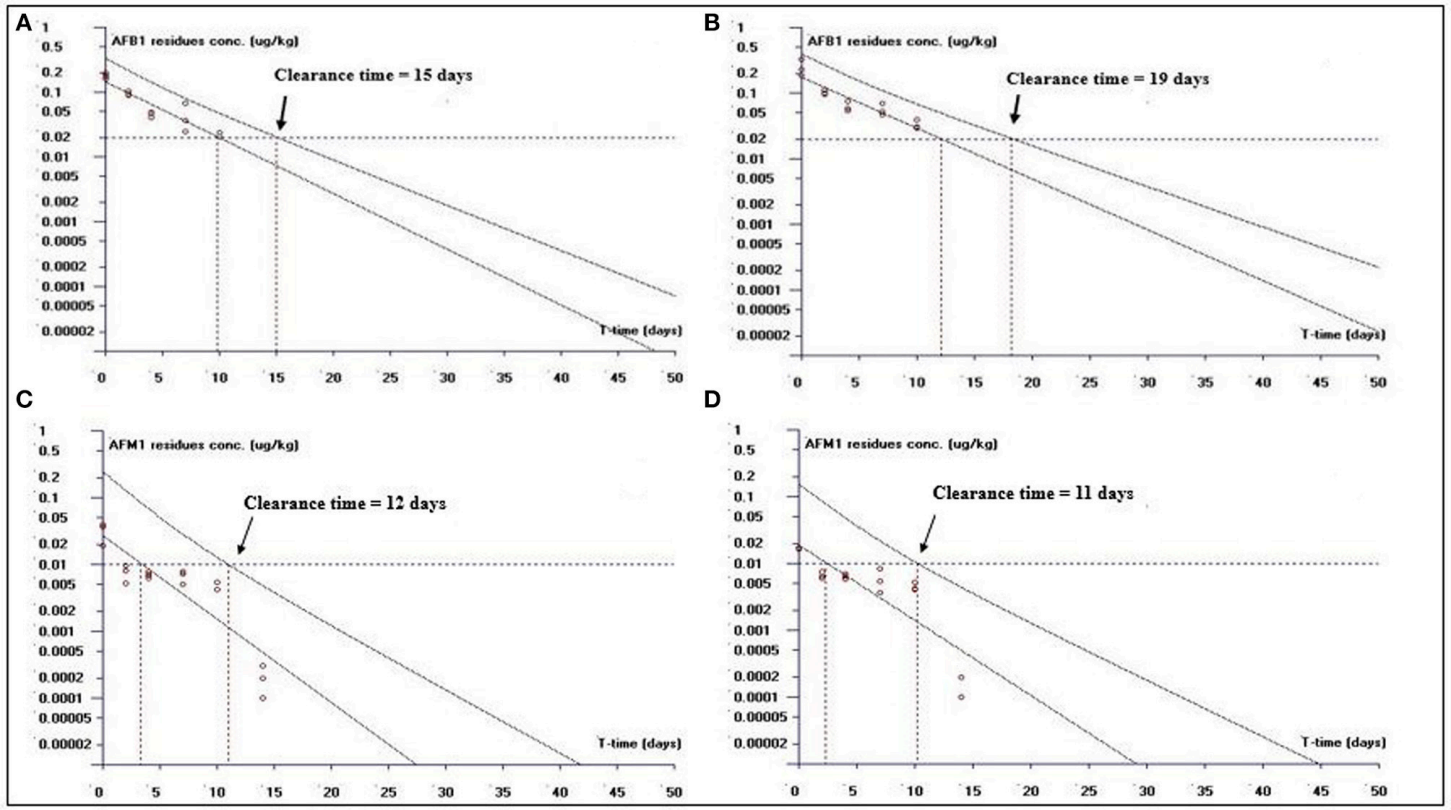
The representative semi-logarithmic plot shows aflatoxin B1 (AFB1) and aflatoxin M1 (AFM1) residue concentrations for broiler **kidney tissues vs. time**, with the one-sided 95% upper tolerance limit in **AFB1-fed group; (A)** AFB1 residues **(C)** AFM1 residues and in **curcumin**+**AFB1-fed group; (B)** AFB1 residues **(D)** AFM1 residues. Small circles represent the residue concentrations of AFB1 and AFM1 for individual broiler chicken. The clearance time calculation based on the limit of quantification (LOQ; 0.02 μg/kg for AFB1 and 0.01 μg/kg for AFM1).

**Figure 3:**
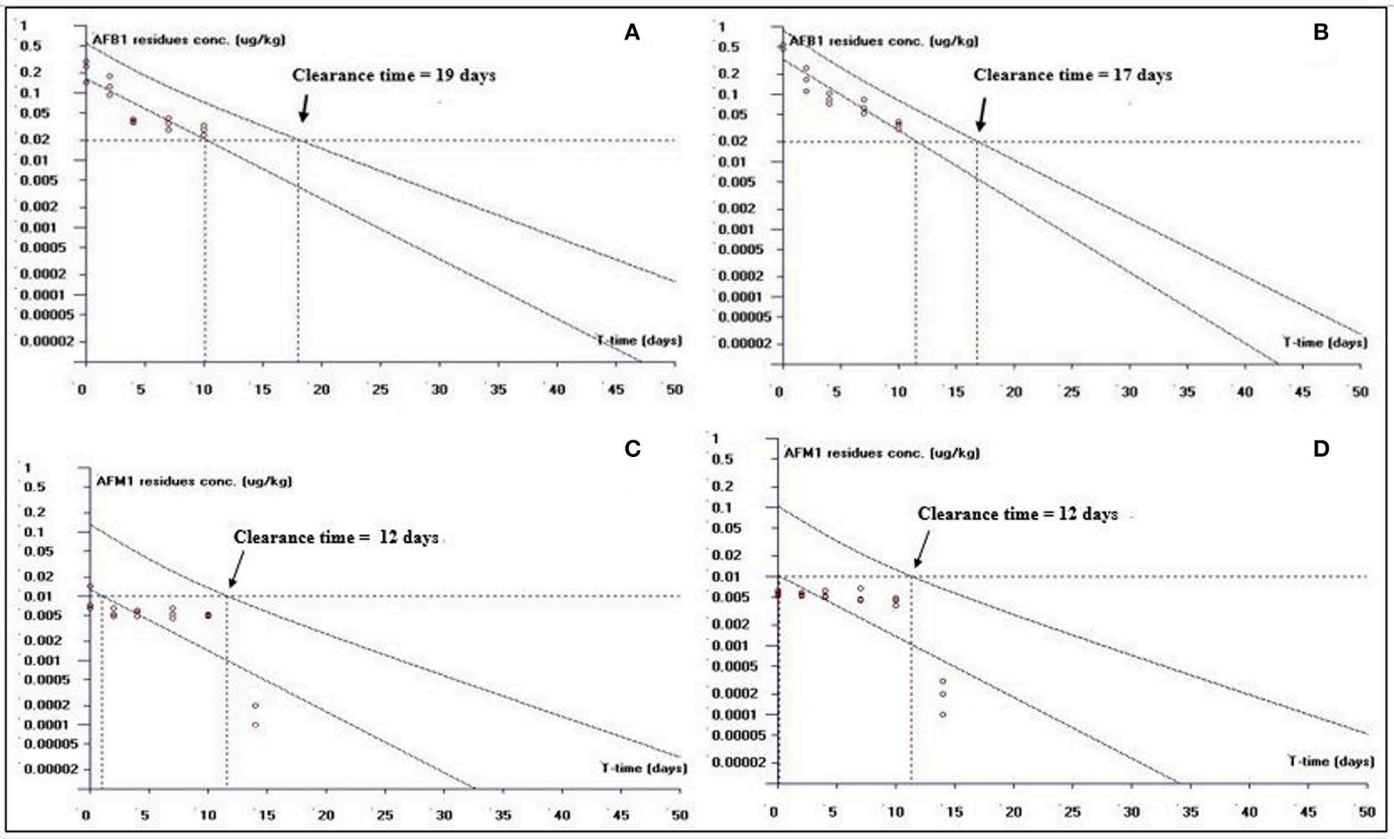
The representative semi-logarithmic plot shows aflatoxin B1 (AFB1) and aflatoxin M1 (AFM1) residue concentrations for broiler **muscles tissues vs. time**, with the one-sided 95% upper tolerance limit in **AFB1-fed group; (A)** AFB1 residues **(C)** AFM1 residues and in **curcumin**+**AFB1-fed group; (B)** AFB1 residues **(D)** AFM1 residues. Small circles represent the residue concentrations of AFB1 and AFM1 for individual broiler chicken. The clearance period calculation based on the limit of quantification (LOQ; 0.02 μg/kg for AFB1 and 0.01 μg/kg for AFM1).

This study is an attempt to propose an appropriate clearance time for AFB1 and AFM1 residues. Figure 1 shows clearance time for AFB1 and AFM1 residues from liver tissues. It has been noted that the clearance time for AFB1 is less in AFB1-fed group (11 days; Figure 1A) than curcumin+AFB1-fed group (18 days; Figure 1B). While, the clearance time calculated for AFM1 residues in AFB1-fed group was 11 days (Figure 1C) and 10 days (Figure 1D) in curcumin+AFB1-fed group. The clearance time of AFB1 residues from kidney tissues displayed in Figure 2. A clearance time of 15 and 19 days have been noted for AFB1 residues in AFB1-fed and curcumin+AFB1-fed group, respectively (Figures 2A,B). On the other hand, 11–12 days clearance time has been noted for AFM1 residues from kidney tissues (Figures 2C,D). Compared to curcumin+AFB1-fed group, the clearance time for AFB1 residues was less in AFB1-fed group from liver and kidney tissues. On the contrary, the clearance time is more in curcumin+AFB1-fed group, thus we speculate that curcumin directly influences/inhibited AFB1-biotransformation which results in prolong the clearance time for AFB1 residues in liver and kidney. Figure 3 showed the clearance time of AFB1 and AFM1 residues from muscles tissues. The clearance time noted for AFB1 residues was 19 days in AFB1-fed group (Figure 3A) and 17 days in curcumin+AFB1-fed group (Figure 3B). Surprisingly, same clearance time (12 days) has been noted for AFM1 residues in both AFB1 group and curcumin+AFB1-fed group from muscle tissues.

### Potential significance of estimating clearance time for AFB1 and AFM1 residues from chickens edible tissues in human diets

The present study showed that residues of AFB1 and AFM1 deposited in broilers edible tissues such as liver, kidney, and muscle tissues and the depletion of these residues may take up to 10–19 days. The clearance time of these residues calculated based on LOQ, revealed that AFB1 and AFM1 residues gradually and readily eliminated after the withdrawal of AFB1-contaminated feed. It is speculated that low levels of AFB1 and AFM1 residues (less than the EU MLs) could showed little likelihood of acute toxicity to human beings from eating AFB1 and/or AFM1-contaminated chickens edible tissues. But these residue concentrations are high enough to cause chronic toxic effects in humans if exposed to the contaminated edible tissues for prolonged periods. Therefore, a necessary clearance time as shown in our results for these residues from chicken's edible tissues must be needed in order to ensure chickens meat safety and protect human health. Some previous studies reported the clearance of aflatoxins residues after withdrawal of aflatoxin contaminated feed within 4 days in broilers (Chen et al., 1984) and 7–8 days in layer chickens (Wolzak et al., 1986). Fernandez et al. demonstrated that clearance time depends on ingested dose and duration of AFB1 and stated that 8-day clearance time was not sufficient for the toxin to disappear completely (Fernandez et al., 1994), they found traces of AFB1 and AFM1 in the 5.0 mg/kg AFB1 group on fourth post-intoxication day in broiler and laying hens. Richard et al. detected residues in turkeys until second week after withdrawing aflatoxin from feed (Richard et al., 1986). Similarly, we computationally identified the clearance time based on LOQ for AFB1 and AFM1 residues depletion in liver, kidney, and muscle tissues. The clearance time determined for the residue depletion of AFB1 and AFM1 in our study was longer than the clearance time mentioned in previous studies (Chen et al., 1984; Richard et al., 1986; Wolzak et al., 1986; Fernandez et al., 1994). This difference might be due to the factors such as dietary AFB1 levels, sex, age, type, species of the birds, duration of administration of AFB1, and the difference in statistical methods used for analysis. Hence, the continuous use of AFB1-contaminated chicken's meat should be avoided for prolong periods to prevent chronic toxic effects. In addition, this field study is carried out to evaluate and ensure the safety of chicken's meat and these types of studies are also of great significance for establishing MLs and necessary clearance time for AFB1 and AFM1 residues in chicken's meat. In conclusion, the optimization and validation parameters indicated that the current method is rapid, accurate, sensitive, selective, and an excellent tool for the determination of AFB1 and AFM1 in broilers edible tissues (liver, kidney, and muscles tissues). There was no matrix interference and the method was linear over the concentrations range of AFB1 and AFM1 studied. The necessary clearance time for AFB1 and AFM1 residues before slaughtering of chickens can be considered as the conclusive clearance time to guarantee consumer safety. The current study signifies that the continuous use of AFB1 and/or AFM1-contamined chicken's meat should be avoided although it contains aflatoxin residues below the MLs (2–4 μg/kg, suggested by EU) as it poses a risk for chronic toxic effects. Taken together, the current method was found satisfactory on the basis of the excellent resolution of analytes, accuracy, robust, precision, shorter chromatographic analysis, reduction of solvent use, and safer for technicians, thus it was found to be appropriate for routine analysis of AFB1 and AFM1 residues in chickens edible tissues to secure food safety and control human health problems associated with these residues.

## Author contributions

XZ supervised the whole experiments. IM contributed to paper writing and XC performed the practical work and completed the experiments. RL, HJ, ZG, YY, JL, and PC provided help during the experiments. SH helped in improving language expression.

### Conflict of interest statement

The authors declare that the research was conducted in the absence of any commercial or financial relationships that could be construed as a potential conflict of interest.

## Supplementary material

Supplementary Figure 1 The effect of shaking time on Recovery (%) of AFB1 and AFM1 (mean ± SD, *n* = 5).

Supplementary Figure 2 All the chromatograms were obtained using mobile phase ratio composed of acetonitrile:water (20:80) at a flow rate of 0.2 ml/min, injection volume of 10 μl and at column temperature of 25°C. The horizontal axis shows the time in minutes (min). **(A)** Chromatogram of standard aflatoxin B1 (AFB1) and aflatoxin M1 (AFM1) (concentrations level; 0.04 μg/kg for AFB1 and 0.02 μg/kg for AFM1), **(B)** chromatogram of AFB1 and AFM1 of spiked (concentrations level; 0.04 μg/kg for AFB1 and 0.02 μg/kg for AFM1) liver samples, **(C)** chromatogram of AFB1 and AFM1 of spiked (concentrations level; 0.04 μg/kg for AFB1 and 0.02 μg/kg for AFM1) kidney samples, **(D)** chromatogram of AFB1 and AFM1 of spiked (concentrations level; 0.04 μg/kg for AFB1 and 0.02 μg/kg for AFM1) muscles tissue samples.
