# Supplementary figures and images for: Development of a UPLC-FLD Method for Detection of Aflatoxin B1 and M1 in Animal Tissue to Study the Effect of Curcumin on Mycotoxin Clearance Rates
Source: Front Pharmacol. 2017 Sep 14;8:650. doi: 10.3389/fphar.2017.00650 (PMC5603667; doi:10.3389/fphar.2017.00650)

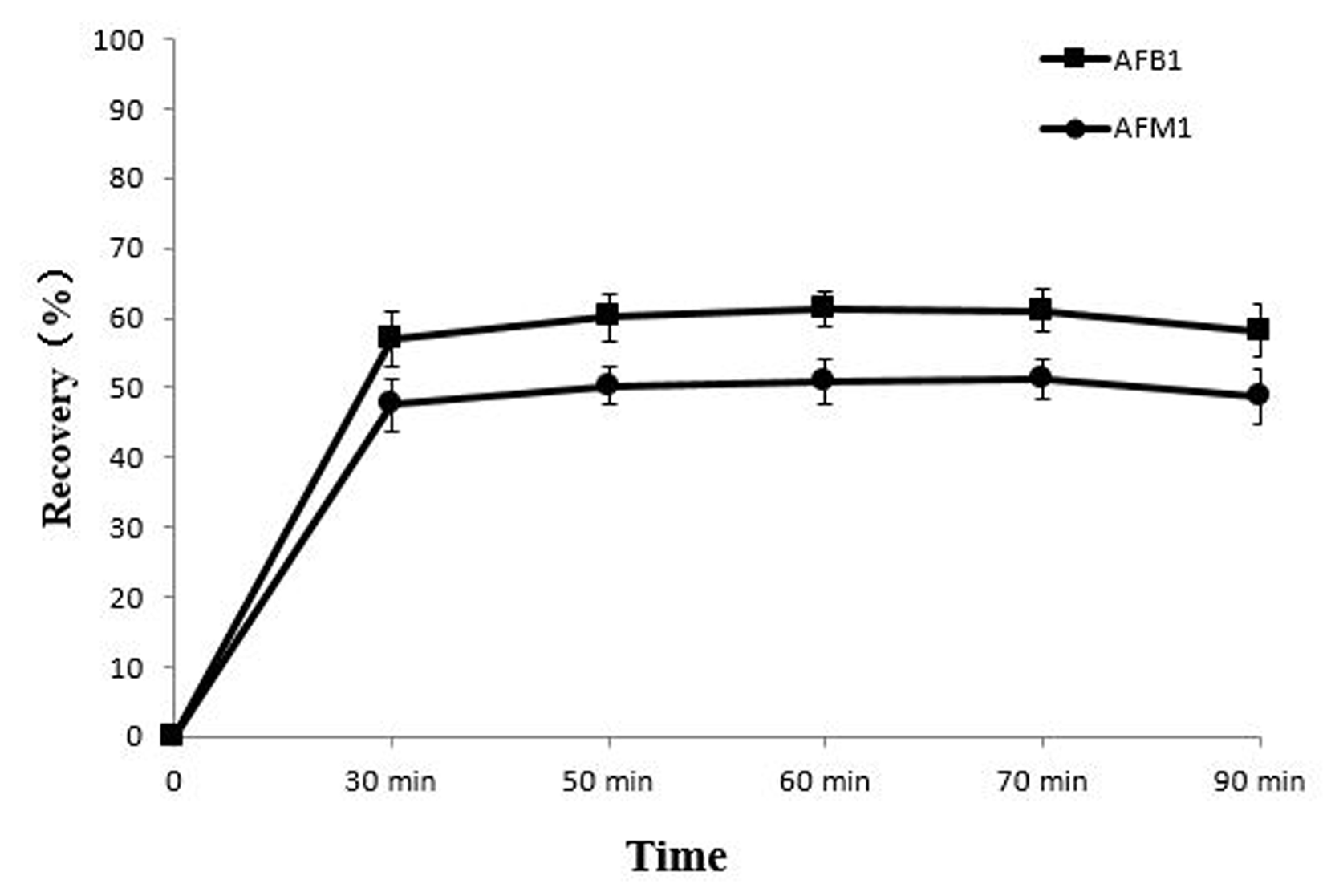

Supplement: Supplementary Figure 1 — The effect of shaking time on Recovery (%) of AFB1 and AFM1 (mean ± SD, n = 5). [file Image1.TIF]

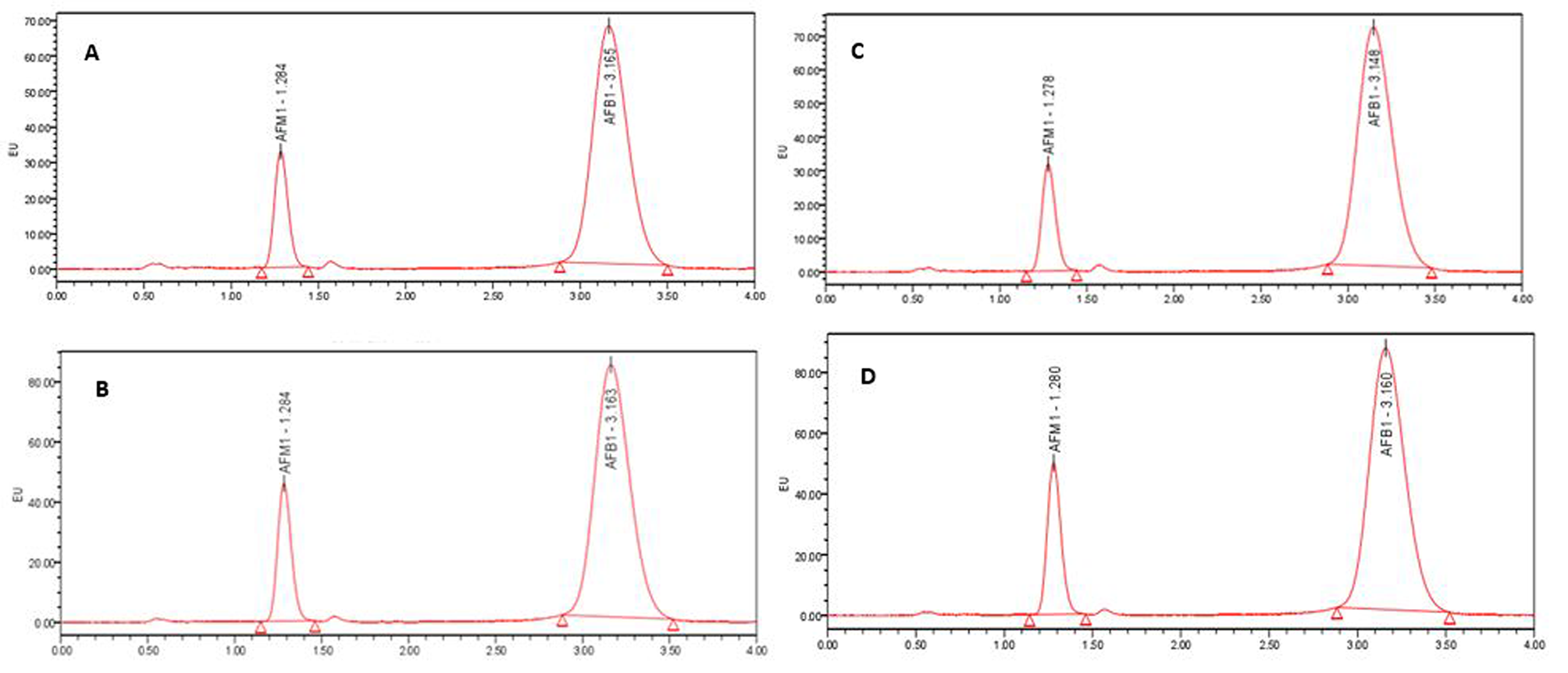

Supplement: Supplementary Figure 2 — All the chromatograms were obtained using mobile phase ratio composed of acetonitrile:water (20:80) at a flow rate of 0.2 ml/min, injection volume of 10 μl and at column temperature of 25°C. The horizontal axis shows the time in minutes (min). (A) Chromatogram of standard aflatoxin B1 (AFB1) and aflatoxin M1 (AFM1) (concentrations level; 0.04 μg/kg for AFB1 and 0.02 μg/kg for AFM1), (B) chromatogram of AFB1 and AFM1 of spiked (concentrations level; 0.04 μg/kg for AFB1 and 0.02 μg/kg for AFM1) liver samples, (C) chromatogram of AFB1 and AFM1 of spiked (concentrations level; 0.04 μg/kg for AFB1 and 0.02 μg/kg for AFM1) kidney samples, (D) chromatogram of AFB1 and AFM1 of spiked (concentrations level; 0.04 μg/kg for AFB1 and 0.02 μg/kg for AFM1) muscles tissue samples. [file Image2.TIF]
